# Phase I/II study testing the combination of AGuIX nanoparticles with radiochemotherapy and concomitant temozolomide in patients with newly diagnosed glioblastoma (NANO-GBM trial protocol)

**DOI:** 10.1186/s12885-023-10829-y

**Published:** 2023-04-15

**Authors:** Emilie Thivat, Mélanie Casile, Juliette Moreau, Ioana Molnar, Sandrine Dufort, Khalide Seddik, Géraldine Le Duc, Olivier De Beaumont, Markus Loeffler, Xavier Durando, Julian Biau

**Affiliations:** 1 Department of Clinical Research, Délégation Recherche Clinique Et Innovation, Centre Jean Perrin, 58 Rue Montalembert, 63011 Clermont-Ferrand, France; 2 INSERM U1240 IMoST, Université Clermont Auvergne, Clermont-Ferrand, France; 3 UMR 501, Centre d’Investigation Clinique, 63001 Clermont-Ferrand, France; 4 Department of Radiation Oncology, Centre Jean Perrin, Clermont-Ferrand, France; 5 NH TherAguix SA, Meylan, France; 6 Oncology Department, Centre Jean Perrin, Clermont-Ferrand, France

**Keywords:** Glioblastoma, Nanoparticles, AGuIX, Nanomedicine, Radiotherapy, Radiosensitization

## Abstract

**Background:**

Despite standard treatments including chemoradiotherapy with temozolomide (TMZ) (STUPP protocol), the prognosis of glioblastoma patients remains poor. AGuIX nanoparticles have a high radiosensitizing potential, a selective and long-lasting accumulation in tumors and a rapid renal elimination. Their therapeutic effect has been proven in vivo on several tumor models, including glioblastoma with a potential synergetic effect when combined with TMZ based chemoradiotherapy, and they are currently evaluated in 4 ongoing Phase Ib and II clinical trials in 4 indications (brain metastases, lung, pancreatic and cervix cancers) (> 100 patients received AGuIX). Thus, they could offer new perspectives for patients with newly diagnosed glioblastoma. The aim of this study is to determine the recommended dose of AGuIX as a radiosensitizer in combination with radiotherapy and TMZ during the concurrent radio-chemotherapy period for phase II (RP2D) and to estimate the efficacy of the combination.

**Methods:**

NANO-GBM is a multicenter, phase I/II, randomized, open-label, non-comparative, therapeutic trial. According to a dose escalation scheme driven by a TITE-CRM design, 3 dose levels of AGuIX (50, 75 and 100 mg/kg) will be tested in phase I added to standard concomitant radio-chemotherapy. Patients with grade IV glioblastoma, not operated or partially operated, with a KPS ≥ 70% will be eligible for the study. The primary endpoints are i) for phase I, the RP2D of AGuIX, with DLT defined as any grade 3–4 NCI-CTCAE toxicity and ii) for phase II, the 6-month progression-free survival rate. The pharmacokinetics, distribution of nanoparticles, tolerance of the combination, neurological status, overall survival (median, 6-month and 12-month rates), response to treatment, and progression-free survival (median and 12-month rates) will be assessed as secondary objectives. Maximum sixty-six patients are expected to be recruited in the study from 6 sites.

**Discussion:**

The use of AGuIX nanoparticles could allow to overpass the radioresistance to the reference treatment of newly diagnosed glioblastomas that have the poorest prognosis (incomplete resection or biopsy only).

**Trial registration:**

Clinicaltrials.gov: NCT04881032, registered on April 30, 2021. Identifier with the French National Agency for the Safety of Medicines and Health Products (ANSM): N°Eudra CT 2020-004552-15. Protocol: version 3, 23 May 2022.

## Background

Glioblastoma (GBM) is the most common malignant brain tumor in adults. Despite the significant improvement with the current standard treatment combining the largest possible resection, followed by concomitant radio-chemotherapy 60 Gy/30 fractions with temozolomide (TMZ) and then adjuvant chemotherapy by several cycles of TMZ [1] the prognosis of these patients remains poor. The median overall survival is 14.6 months (18.8 months, 13.5 months, and 9.4 months for patients with complete resection, partial resection, and biopsy alone, respectively) [2] and the progression-free survival at 6 months is 53.9% [1] with a poorer prognostic for the patient with unresectable GBM [2]. Moreover, GBMs are radioresistant tumors and recurrence occurs in approximately 90% of cases within the radiation fields [3]. Since there are few therapeutic solutions in case of recurrence, a radiosensitization strategy would permit to increase the chances of local control, from the first line of treatment on.

Metal nanoparticles with a high atomic number (Z) have the ability to act as radiosensitizers. They allow improving the efficiency of irradiation, by increasing the absorption of photons emitted by the radiation beam, in tumor cells. The use of nanoparticles could be a promising approach for the local treatment of tumors by external radiotherapy (RT). The radiosensitizing agent evaluated in this study is AGuIX (Activation and Guidance of Irradiation by X-ray) nanoparticles, a gadolinium chelated-polysiloxane based-nanoparticles administered intravenously [4]. This nanoparticle is a theranostic agent with very high radiosensitizing properties as well as contrast agent properties, thanks to the presence of gadolinium. The nanoparticles were tested on different orthotopic tumor models of the central nervous system (gliosarcoma [5], glioblastoma [6] and melanoma brain metastases [4]) and on mice with heterotopic tumors. Thanks to the EPR (Enhanced Permeability and Retention) effect [7], a long accumulation and retention of nanoparticles in tumor tissues is observed and they are still detectable by MRI 24 h after their intravenous administration. The nanoparticles are characterized by rapid clearance by normal tissue, high tumor-to-healthy tissue affinity ratio, with no evidence of extravasation into the brain tissue. In vivo studies have demonstrated such efficacy when combining AGuIX nanoparticles with radiation in animals with GBM [5, 8, 9] lung tumors [7], head and neck tumors [10], pancreatic tumors [11], melanoma [12] and multifocal brain melanoma metastases [4] with a survival benefit with the combination of AGuIX nanoparticles and RT, compared to RT alone. In an orthotopic glioma rat model, Dufort et al. demonstrated the selective accumulation of AGuIX in high-grade glioma as well as the potential survival benefits when combined with standard of care TMZ and radiotherapy [13].

The first-in-human phase I NANO-RAD (NCT02820454) evaluated the tolerance and the maximum tolerated dose of the intravenous injection of AGuIX nanoparticles, (15, 30, 50, 75 or 100 mg/kg) in combination with whole brain radiotherapy in patients with multiple brain metastases not suitable for stereotactic radiotherapy (from primary cancers of melanoma type *n* = 6, non-small cell lung cancer *n* = 6, colon cancer *n* = 1, and breast cancer *n* = 2). No dose-limiting toxic effects were observed up to AGuIX 100 mg/kg. This clinical trial demonstrated a good tolerance of the intravenous injection of AGuIX nanoparticles, and a rapid elimination by the kidney in correspondence with preclinical results. Among the 29 SAEs, four were considered possibly related to AGuIX and were delayed effects of RT, possibly enhanced by AGuIX [14]. Moreover, this clinical trial also demonstrated (i) the selective accumulation of AGuIX in brain metastases from different types of primary tumors due to the EPR effect, with a persistence in the tumor lasting few days after injection, (ii) a dose-correlated uptake of AGuIX in brain metastases, (iii) a dose-correlated tumor volume reduction and (iv) a clinical benefit for 13 patients over 14 when combination of AGuIX with radiotherapy [15].

Four clinical phase Ib and/or II trials are currently underway with AGuIX, with more than 100 patients already injected with AGuIX nanoparticles. The NANORAD 2 (NCT03818386) and the NANOBRAINMETS (NCT04899908) trials are randomized phase II trials evaluating the efficacy of the combination of intravenous injections of AGuIX nanoparticles (100 mg/kg) with whole brain radiotherapy or stereotactic radiosurgery/radiotherapy, respectively, in the treatment of brain metastases. The NANOSMART trial (NCT04789486) is a phase I/II trial evaluating the safety and the efficacy AGuIX in combination with stereotactic magnetic resonance-guided adaptive radiation therapy for centrally located lung tumors and locally advanced pancreatic cancers, and the NANOCOL trial (NCT03308604) is a phase Ib evaluating AGuIX in combination with radio-chemotherapy in advanced cervical cancer.

According to preclinical and clinical results, the AGuIX nanoparticles could be a promising approach to increase the effectiveness of the standard of care (TMZ and RT) in GBM patients.

Our phase I/II trial aims to test the combination of AGuIX nanoparticles with radio-chemotherapy and concomitant TMZ in patients with newly diagnosed GBM with incomplete resection.

## Methods / design

### Study design

NANO-GBM trial is a multicenter, phase I/II, randomized, open-label, non-comparative, therapeutic study.

The phase I part consists of a dose escalation with 3 dose levels of AGuIX: 50, 75 and 100 mg/kg, driven by a Time-to-event Continuous Reassessment Method (TITE-CRM) [16] with a dose-toxicity relationship given by a one-parameter power model and a priori risks of 5%, 10% and 25% for the 3 doses, respectively. The first patient will receive the first dose level, and the next cohorts will consist of 1 patient at a time, with a minimum of 6 treated at the recommended dose and a maximum of 12 patient treated in phase I.

The phase II part consists of a randomized open-label study. Patients will be randomized at baseline via a central system on eCRF, in a 2:1 ratio stratifying for the site and the age: one third of patients in the reference arm treated with radio-chemotherapy with concomitant TMZ (STUPP protocol), and two thirds in the experimental arm combining radio-chemotherapy with concomitant TMZ with the administration of AGuIX nanoparticles at the recommended dose determined in the phase I part. The randomization sequence is known only to the statistician. For the experimental arm, a single-stage Fleming design will be used. While the design does not allow for a formal comparison between the two arms, it may reinforce the value of the conclusions on the potential efficacy of the experimental treatment by validating the hypothesis on the control arm.

An interim analysis will be performed without interruption of inclusions for the independent data monitoring committee (IDMC) review, after 20 patients are treated in the experimental arm at the recommended dose (i.e. approximately 30 patients in phase II).

A maximum of sixty-six patients are expected to be enrolled: 12 patients in phase I, of which (minimum) 6 are treated at the recommended dose, 34 additional patients in the phase II experimental arm and 20 patients in the phase II reference arm. The study was started in March 2022 with a 2 years enrollment period and an estimated completion date by March 2026. This study has been registered on Clinicaltrials.gov (NCT04881032).

### Coordination and participating institutions

The Centre Jean PERRIN is the sponsor and is responsible for coordination, trial management, data management, trial monitoring and statistical analysis. This multicenter study will be conducted in 6 sites in France.

The list of the study sites is available on https://clinicaltrials.gov/ct2/show/NCT04881032

### Study objectives and endpoints

#### Mains objectives and endpoints

The primary objectives of the NANO-GBM study are i) in the phase I part, to determine the recommended dose of AGuIX in combination with radiotherapy and TMZ during the concurrent radio-chemotherapy period for phase II (RP2D); ii) in the phase II part, to estimate the efficacy of the combination radio-chemotherapy + AGuIX (at the RP2D).

The first primary endpoint (phase I) is the RP2D of AGuIX nanoparticles corresponding to the highest dose tested for which the percentage of dose-limiting toxicities (DLT) is less than 33%. DLT is defined as any grade 3–4 toxicity according to the NCI-CTCAE classification v5.0, except for alopecia, nausea and vomiting, or fever, which can be managed by symptomatic treatment. Only toxicities occurring during the concomitant radio-chemotherapy (i.e. during 6 weeks) will be considered for DLT assessment.

The second primary endpoint (phase II) is 6-month progression-free survival rate (6-month PFS) to estimate the efficacy of the combination of radio-chemotherapy with AGuIX. Disease assessment throughout the study will be based on RANO (Response Assessment in Neuro-Oncology) criteria that take into account imaging progression (MRI) and also clinical status and level of steroid therapy [17].

#### Secondary objectives

The secondary objectives are to evaluate:

- The pharmacokinetics parameters (AUC, Tmax et Cmax) of AGuIX nanoparticles (phase I only) will be measured on blood samples and urinary excretion at D0, D7, D14
- The distribution of nanoparticles and the sparing of healthy tissue by MRI (W0 and D14)
- The tolerance of the combination: acute (< 90 days) and late toxicity as well as changes in dose and spread of radiotherapy (phase I and II) will be graded according to the Common Terminology Criteria for Adverse Events (CTCAE, version 5.0). Reporting of serious adverse events and suspected unexpected serious adverse reaction will be carried out according to the local regulations.
- The neurological status will be evaluated by clinical assessment and Mini-Mental State Examination (MMSE)
- Overall survival (median, 6-month and 12-month rates), response to treatment, and progression-free survival (median and 12-month rates) according to RANO criteria (phase II)

#### Exploratory objective

A FFPE block of the tumor will be collected for each patient, unless they oppose it on the informed consent form. This block will be stored by the study sponsor and will be used to conduct exploratory translational research. The objective is to study potential predictive biomarkers and exploration of the tumor microenvironment.

### Participant eligibility

The inclusion and non-inclusion criteria are presented in Table 1.

**Table 1:** Section criteria

Inclusion criteria	Non-inclusion criteria
Histological diagnosis of grade IV glioblastoma (biopsy or partial surgery)	History of cerebral radiotherapy
Patient not operated or in partial excision	History of chemotherapy (including carmustine (Gliadel®) implants) or immunotherapy (including vaccination)
Karnofsky performance status ≥ 70%	Any contraindication to TMZ listed in the multidisciplinary consultation meeting
Age ≥ 18 years and < 75 years	History of major bowel resection that may affect oral drug absorption in the judgment of the investigator
Life expectancy ≥ 6 months	Diagnosed inflammatory bowel disease (Crohn's disease or ulcerative colitis)
Platelets ≥ 100 000/mm^3^	Diarrhea ≥ CTCAE grade 2 (regardless of cause)
PNN ≥ 2000/mm^3^	Current or recent treatment with another investigational drug or participation in another therapeutic clinical trial (within 30 days of inclusion)
Hemoglobin ≥ 10 g/dL	History of other cancer within 5 years prior to inclusion except basal cell carcinoma of the skin and carcinoma in situ of the cervix
Creatinine < 1.5 times the upper normal limit or Cockcroft-Gault clearance ≥ 50 mL/min	Pregnant or breastfeeding women
Liver function (GGT, PAL, AST, ALT, bilirubin) < 1.5 times the upper normal limit	Contraindication to MRI or gadolinium injection
For patients receiving corticosteroid therapy, corticosteroid therapy must be at a stable or decreasing dose for at least 14 days prior to inclusion	History of severe anaphylactic reactions due to the injection of gadolinium-based contrast material (Dotarem®, etc.)
Patient able to swallow and maintain oral therapy	Patient under guardianship or curatorship
Negative serum pregnancy test within 7 days prior to first administration of therapy for women	History of nephropathy
Women of childbearing potential and men whose partners are of childbearing potential must agree to use an approved method of contraception for themselves or their partners for the duration of treatment and at least 6 months after the last study treatment	Psychological disorder or social or geographical reasons that could compromise the medical follow-up of the trial or compliance with the treatment
Obtaining signed informed consent from the patient	
Patient enrolled in a social security plan	

Patients with grade IV glioblastoma, not operated or partially operated, with a KPS ≥ 70% will be eligible for the study. However, patients with a history of cerebral radiotherapy, chemotherapy or with a contraindication to TMZ or MRI or gadolinium injection, will not be eligible for the trial.

### Intervention

#### Experimental group

**Fig. 1:**
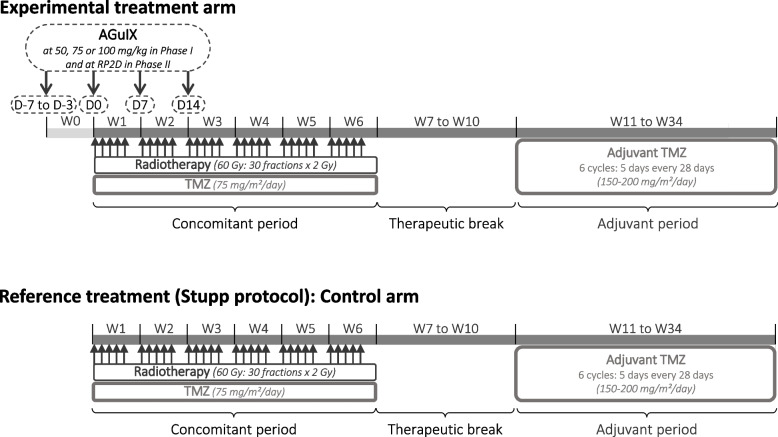
Overview of the treatment period of the NANO-GBM study

Patients in the phase I and phase II experimental arms will be treated with concomitant TMZ radio-chemotherapy (STUPP protocol) in combination with AGuIX nanoparticles during the concomitant radio-chemotherapy period (Fig. 1).

Nanoparticles AGuIX (experimental treatment) provided by NH TherAguix, will be administered in 4 intravenous injections. The first one will be administered 3 to 7 days before the start of the radio-chemotherapy and at least 48 h after dosimetry MRI. The next three injections will be administered during the radio-chemotherapy at the first day of the week 1, week 2 and week 3. The injection will be performed 4 h (± 1 h) before RT and 3 h before TMZ. For phase I, the dose administered at each injection will be 50, or 75 or 100 mg/kg (tested doses). For phase II, the dose administered at each injection will be the dose recommended after phase I.

#### Control group

Patients in the phase II reference arm will be treated with the standard STUPP protocol: RT at a dose of 60 Gy (30 fractions/6 weeks) and concomitant treatment with daily oral TMZ (75 mg/m^2^/day) from the first day of RT until the end of RT. Then, adjuvant TMZ will be reintroduced 4 weeks after the end of RT according to a 5-days schedule every 28 days (150 mg/m^2^/day for the first cycle and then at 200 mg/m^2^/day in the absence of toxicity).

### Study procedures and participant timeline

The overview of study assessments and procedures is presented in Table 2**.**

**Table 2:** Data collection schedule

	**Screening / inclusion**		**Concomitant chemoradiotherapy phase**						**Therapeutic break**				**Adjuvant phase**							
Timepoint													**cycle 1**				**cycle 2**			
Week		**W0**	**W1**	**W2**	**W3**	**W4**	**W5**	**W6**	**W7**	**W8**	**W9**	**W10**	**W11**	**W12**	**W1 3**	**W1 4**	**W1 5**	**W16**	**W1 7**	**W18**
Day	**(-28 à -7)**	**at -7 to -3**	**0**	**7**	**14**	**21**	**28**	**35**	**42**	**49**	**56**	**63**	**70 ± 4d**	**77**	**84**	**91**	**98 ± 4d**	**105**	**112**	**119**
Informed consent	✓																			
Medical history and demographics	✓																			
Initial histology / centralized review (FFPE tumor block)	✓																			
Previous/concomitant treatments (including corticosteroids)	✓		throughout the treatment																	
Evaluation of adverse events			throughout the treatment																	
Radiological evaluation MRI (disease)	✓(according to standard practice)												✓							
MRI dosimetry	✓(-15 à -5)																			
MRI post injection AGuIX (1 h after injection)^h^		✓^**h**^			✓^**h**^															
Examination neurological (MMSE)	✓												✓							
Clinical examination, weight	✓	✓^**h**^	✓	✓	✓	✓	✓	✓					✓				✓			
Vital signs^a^	✓	✓^**h**^	✓	✓	✓	✓	✓	✓					✓				✓			
KPS Performance Index	✓	✓^**h**^	✓	✓	✓	✓	✓	✓					✓				✓			
Serum pregnancy test^g^	✓				✓			✓					✓				✓			
Hematology^b^	✓	✓^**h**^	✓	✓	✓	✓	✓	✓					✓				✓			
Fasting biochemistry^c^	✓	✓^**h**^	✓	✓	✓	✓	✓	✓					✓				✓			
Urine analysis^d^	✓	✓^**h**^	✓	✓	✓	✓	✓	✓					✓				✓			
Pharmacokinetics^f^			✓^f^	✓^f^	✓^f^															
Radiotherapy			**2 Gy x 5d / week pour 6 weeks**																	
Temozolomide			**75 mg/m**^**2**^** daily intake D0 à D42**										**5D**^**e**^				**5D**^**e**^			
AGuIX (injection IV)^i^		**D-7 to D- 3**^**j,h**^	**D0**^**h**^	**D7**^**h**^	**D14**^**h**^															
survival assessment			throughout the study																	

	**Adjuvant phase**																**End of treatment visit**	**Follow-up to progression**	**Follow-up of survival**
Timepoint	**cycle 3**				**cycle 4**				**cycle 5**				**cycle 6**					**every 3 months for 18 months after V(W35) then every 6 months**	**every 3 months for 18 months after V(W35) then every 6 months**
Week	**W19**	**W20**	**W21**	**W22**	**W23**	**W24**	**W25**	**W26**	**W27**	**W28**	**W29**	**W30**	**W31**	**W32**	**W33**	**W34**	**W35**		
Day	**126 ± 4d**	**133**	**140**	**147**	**154 ± 4d**	**161**	**168**	**175**	**182 ± 4d**	**189**	**196**	**203**	**210 ± 4d**	**217**	**224**	**231**			
Informed consent																			
Medical history and demographics																			
Initial histology / centralized review (FFPE tumor block)																			
Previous/concomitant treatments (including corticosteroids)																			
Evaluation of adverse events																			
Radiological evaluation MRI (disease)					✓												✓	✓	
MRI dosimetry																			
MRI post injection AGuIX (1 h after injection)^h^																			
Examination neurological (MMSE)					✓												✓	✓	
Clinical examination, weight	✓				✓				✓				✓				✓	✓	
Vital signs^a^	✓				✓				✓				✓				✓		
KPS Performance Index	✓				✓				✓				✓				✓	✓	
Serum pregnancy test^g^	✓				✓				✓										
Hematology^b^	✓				✓				✓				✓				✓		
Fasting biochemistry^c^	✓				✓				✓				✓				✓		
Urine analysis^d^	✓				✓				✓				✓				✓		
Pharmacokinetics^f^																			
Radiotherapy																			
Temozolomide	**5D**^**e**^				**5D**^**e**^				**5D**^**e**^				**5D**^**e**^						
AGuIX (injection IV)^i^																			
survival assessment																			✓

^a^vital signs include heart rate measurement, systolic and diastolic blood pressure, temperature, pulse

^b^hemoglobin, CBC platelet

^c^glucose, sodium, potassium, magnesium, calcium, total protein, creatinine, urea, AST, ALT, alkaline phosphatase, GGT, total bilirubin

^d^proteinuria

^e^TMZ 150—200 mg/m^2^/d D1 at D5 every 28 days (the daily dose of 150 mg/m^2^ for the first cycle will be increased to 200 mg/m^2^ for the 2nd cycle in the absence of significant toxicity)

^f^pharmacokinetics performed only for phase I patients: blood sampling 7 ml before injection, 15 min, 30 min, 1 h (before MRI), 2 h, 3 h, 4 h (before the beginning of radiotherapy), 6 h and 24 h post injection + urine collection during 6 h

^g^for patients of childbearing age

^h^only for patients receiving the experimental treatment: Phase I + Phase II experimental arm

^i^experimental treatment: for phase I patients (dose of 50 or 75 or 100 mg/kg according to the TITE CRM) and for patients in the experimental arm of phase II (at the dose recommended by phase I) At S1J0, S2J7 and S3J14 the injection will be performed 4 h ± 1 h before the radiotherapy session

^j^injection performed 3 to 7 days before the start of radiochemotherapy, and at least 2 days after the MRI dosimetry

### Statistical analysis

#### Sample size

A maximum of 12 patients will be treated in phase I (sample size obtained by simulation, and in agreement with the bound obtained by Cheung’s formula, 2013), minimum 6 patients to be treated at the recommended dose, who will then be included and analyzed in phase II.

The phase II trial consists of a randomized study with a 2:1 ratio in favor of the experimental arm. For the experimental arm, a single-stage Fleming design will be used, based on an acceptable success rate of 70% and a 50% unacceptable success rate. In order to limit the one-sided alpha risk (of wrongly concluding that the treatment is effective) to 5% and the beta risk (of wrongly failing to conclude that the treatment is effective) to 20%, the minimum number of patients to include is 37. The treatment will be considered effective (and therefore the phase II positive) as soon as 24 successes out of 37 are obtained. In this case, the estimate of the probability of success will be of around 65%, with a 90% confidence interval (90% CI) of 51.4%-76.7% (Jeffreys interval).

It was decided to include 40 patients in the experimental arm, to compensate for a rate of about 10% of missing data (all reasons combined). Therefore, the number of patients in the reference arm will be 20.

Consequently, the total number of patients to be included is 66 (maximum): 12 patients in phase I, of which (minimum) 6 were treated at the recommended dose, 34 additional patients in the experimental arm of phase II, and 20 patients in the reference arm of phase II.

### Data analysis

Statistical analysis will be realized on modified intention-to-treat (mITT) population, i.e. patients who received at least 1 injection of AGuIX nanoparticles.

The primary endpoint of phase I, the RP2D of AGuIX nanoparticles, will be determined as the highest dose tested for which the percentage of dose-limiting toxicities (DLT) is less than 33%. DLT percentage will be calculated for each tested dose. Progression-free survival, progression-free survival rate at 6 months and 12 months, and overall survival at 6 months and 12 months will be estimated by the Kaplan–Meier method in both arms. The primary endpoint of phase II, 6-month PFS in the experimental arm, will be calculated with its 90% CI, and the result will be interpreted according to the Fleming method.

Missing data will not be replaced. The statistical significance threshold is set at 5%. Statistical analyses will be performed using R software.

### Data management and monitoring

The data collected for the study will be recorded in an eCRF (Ennov Clinical software) by each investigating center. The staff with access to the data will be the investigators, the clinical research associates, the project leaders and the biostatisticians. They are authorized professionals and are subject to professional secrecy. The investigator will ensure the accuracy, completeness, and consistency of the pseudonymized patient data recorded and of the provision of answers to data queries.

Regular monitoring reviews will be performed by a clinical research associate mandated by the sponsor. The objectives will be to ensure the proper conduct of the study in each center, the recording of the data generated in writing, their documentation, recording and reporting, in accordance with the legislative and regulatory provisions in force. The monitoring reports will ensure traceability.

### Independent data monitoring committee (IDMC)

An independent monitoring will be responsible for reviewing the safety data of treated patients. The committee will meet at the end of phase I and will be able to validate the recommended dose retained, recommend the continuation of the trial in phase II, its interruption, or its modification depending on the adverse events which occurred during the trial.

The committee will meet a second time during phase II, after 20 patients have been treated at the RP2D in the experimental arm (end of the TDL evaluation period) to confirm the choice of the recommended dose.

This committee will consist of 3 members who are not involved in patient recruitment or trial conduct and will include at least 1 radiotherapist and 1 oncologist.

### Trial status

The NANO-GBM clinical trial is currently recruiting. Patient enrollment began in March 2022 and is expected to end in March 2024.

## Discussion

The NANO-GBM trial is the first study to test the association of intravenous nanoparticles with radio-chemotherapy (STUPP standard protocol) in GBM patients. This trial aims to determine the RP2D of AGuIX nanoparticles and evaluate the efficacy of the combination of AGuIX nanoparticles with radio-chemotherapy and concomitant TMZ in patients with newly diagnosed GBM (with incomplete resection).

The approach to improve the standard treatment of newly diagnosed GBM is based on an optimization of RT, as surgery is always maximal and functional and remains a major prognostic factor, and given that TMZ doses cannot be increased due to associated toxicities.

The accumulation of AGuIX nanoparticles in tumors followed by the interaction between AGuIX nanoparticles and the X-rays, combined with an elimination of nanoparticles in healthy tissues, would allow increasing the effectiveness of RT at the tumor site, without increasing the prescribed X-ray dose. Moreover, it has been demonstrated in preclinical tumor models but also during the First in Human phase I trial that the drug is not active except at the irradiated tumor area: the first clinical data report a good tolerance of a single intravenous AGuIX. The use of AGuIX nanoparticles could therefore allow to overpass the radioresistance to the reference treatment (STUPP protocol) of newly diagnosed unresectable or partially resected GBM while keeping the same level of sparing for healthy tissues. It represents a promising method to improve care of the poorest prognosis patients.

With the use of AGuIX nanoparticles, we expect an improvement of the 6-month progression-free survival rate by 20% for these patients who have the poorest prognosis, without an increase of the delivered total dose of RT and without increase of toxicity in healthy tissues. An improvement in their neurological symptoms linked to the expected response on the residual tumor or in place by increased radiosensitivity is also expected (reduction in signs of intracranial hypertension by reduction in the mass effect or reduction in neurological symptoms).

## Data Availability

Not applicable.

## Abbreviations

DLT: Dose-Limiting Toxicity
EPR: Enhanced Permeability and Retention
GBM: Glioblastoma
MTD: Maximum Tolerated Dose
RANO: Response Assessment in Neuro-Oncology
RP2D: Recommended Phase II dose
TITE-CRM: Time-to-event CRM
TMZ: Temozolomide
